# Immediate Reduction in Left Ventricular Contractility After Mitral Transcatheter Edge‐to‐Edge Repair Is Associated With Lower Rates of Heart Failure Hospitalizations

**DOI:** 10.1161/JAHA.124.037545

**Published:** 2025-02-26

**Authors:** Ke‐Wei Chen, Ju‐Hsin Chang, Agata Sularz, Gerardo V. Lo Russo, Ghasaq Saleh, Shih‐Sheng Chang, Chia‐hao Liu, Mohamad Alkhouli

**Affiliations:** ^1^ Department of Cardiovascular Medicine Mayo Clinic Rochester MN USA; ^2^ Division of Cardiology, Department of Internal Medicine China Medical University Hospital Taichung Taiwan; ^3^ Department of Anesthesiology China Medical University Hospital Taichung Taiwan; ^4^ Department of Clinical Sciences and Community Health, Cardiovascular Section University of Milan Milan Italy; ^5^ School of Medicine, College of Medicine, China Medical University Taichung Taiwan

**Keywords:** heart failure readmission, left ventricular ejection fraction, mitral valve transcatheter edge‐to‐edge repair, mortality, Valvular Heart Disease, Heart Failure

Mitral valve transcatheter edge‐to‐edge repair (TEER) is an increasingly used treatment in patients with severe mitral regurgitation who remain symptomatic despite optimal medical therapy. TEER has been shown to improve survival and reduce heart failure readmission rates even in patients with very low cardiac output.^1^ Limited evidence suggests that hemodynamic improvement after TEER occurs in distinct phases.^2^, ^3^ Typically, after clip deployment, there is a sudden increase in the left ventricular (LV) afterload that may unmask preexisting impairment of myocardial contractility.^4^ The left ventricle exhibits various acute responses to TEER; the consequence of which is not fully understood. For instance, previous reports suggest that a decrease in LV ejection fraction (LVEF) on postoperative echocardiography correlates with improved outcomes.^5^ Herein, we evaluate the intraoperative changes in LVEF as potential predictors of mortality and heart failure hospitalization in patients undergoing TEER.

We included patients who underwent TEER and had adequate intraoperative transesophageal echocardiography images to assess LVEF intraoperatively (before and after TEER). The Mayo Clinic Institutional Review Board approved this study and waived the requirement for patient consent, as it was classified as a minimal‐risk observational research study. The data supporting the findings of this study can be obtained from the corresponding author upon reasonable request. We defined LVEF reduction ratio as the ratio of the difference between baseline LVEF and post‐TEER LVEF indexed by baseline LVEF. We stratified patients into 2 groups according to the immediate change in their LVEF: group 1, with increase in LVEF or with a reduction ratio of <10%, and group 2, with LVEF reduction ratio ≥ 10%. Baseline characteristics were compared with Mann–Whitney *U* test for continuous variables and Pearson's chi‐square for categorical variables. The primary outcome was a composite of all‐cause mortality and heart failure admissions and was assessed via a survival analysis using the Kaplan–Meier method. Adjusted hazard ratios (aHR) were estimated using Cox regression analysis.

A total of 134 patients were included. The mean age was 78.17 (interquartile 74.1–86.0), and majority were male (61.9%). There were 102 patients in group 1 and 32 patients in group 2. At baseline, group 1 had a significantly higher LVEF (53.2 *±* 14.6 versus 42.8 *± 11.6*) and lower cardiomyopathy rates (68.7% and 46.1%) than group 2. The median follow‐up was 362 days and 82.84% of patients were alive at 1 year. Heart failure readmission rate was 12.69% at 1 years. There was no significant difference in mortality between the 2 groups. The aHR for the LVEF reduction ratio *≥* 10% group was 0.31 (95% CI, 0.11–0.90; *P =* 0.032), after adjusting for mean left atrial pressure and diuretic use (Figure, Left panel). Within group 2, 84.4% of the patients underwent echocardiographic follow‐up, which showed that 31.25% of group 2 patients achieved or exceeded the baseline LVEF. The degree of intraoperative reduction in LVEF is highly correlated with the extent of recovery observed during postoperative follow‐up echocardiography. Patients who experience a greater decline in LVEF during surgery tend to exhibit more pronounced recovery postoperatively. (Figure, Right panel).

**Figure 1:**
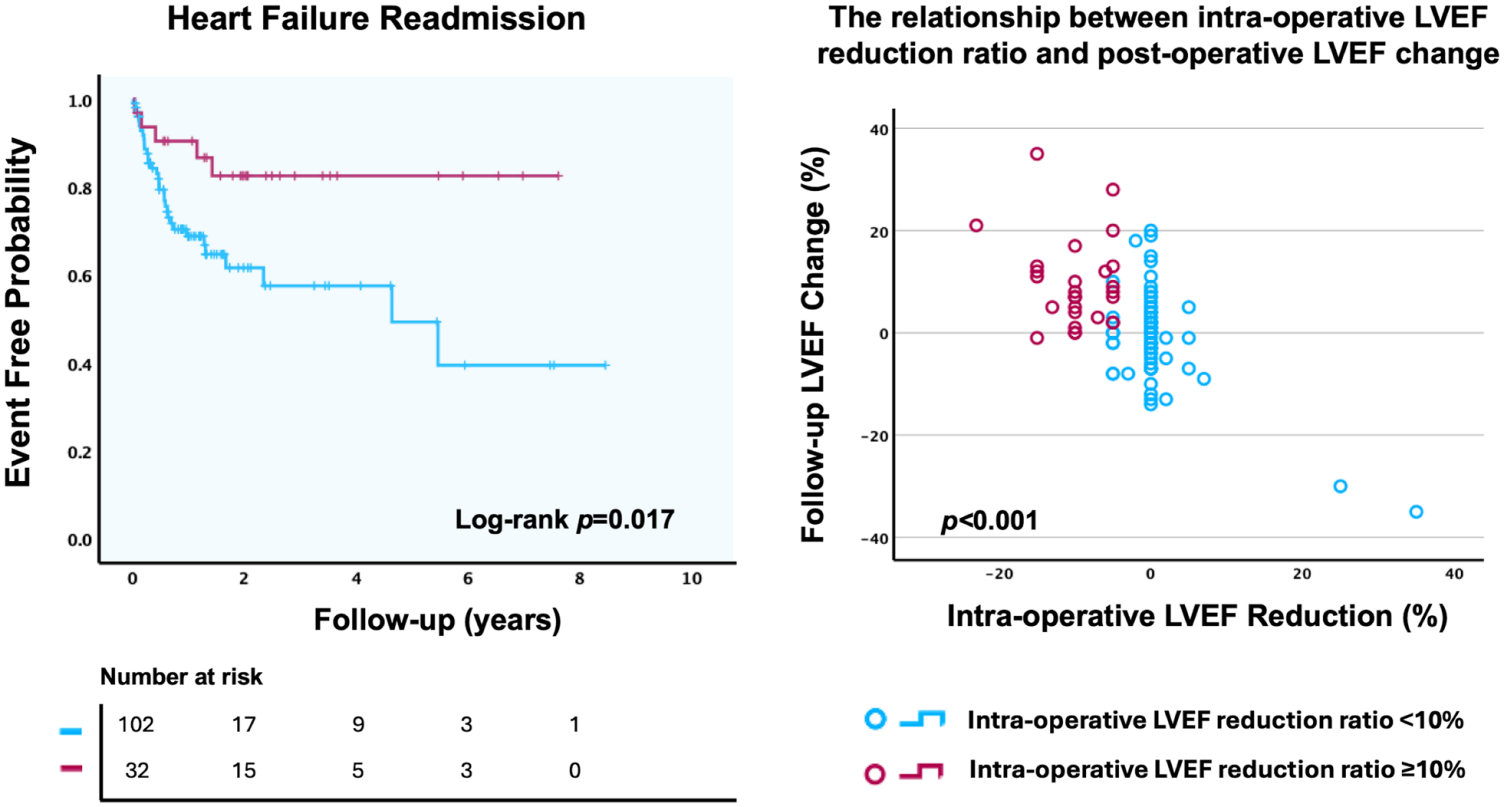
Impact of LVEF reduction on heart failure hospitalization and longitudinal LVEF trends after TEER. Left: Cumulative incidence of heart failure hospitalization following TEER according to LVEF reduction ratio. Right: trend of mean LVEF changes between groups. LVEF indicates left ventricular ejection fraction; and TEER, mitral valve transcatheter edge‐to‐edge repair.

Our focused analysis shows that, in patients undergoing TEER, a less pronounced reduction in intraoperative LVEF correlates with higher heart failure readmission rates. Successful TEER leads to an apparent discrepancy of LV afterload before and after the procedure. This effect is caused by sudden loss of left atrial unloading. Immediate increases in LV afterload may contribute to an immediate decline in LVEF, and it is possible that this may be a marker of successful TEER. Despite a significant intraoperative LVEF decline in Group 2, follow‐up echocardiography shows this is likely temporary and not indicative of permanent damage. The key limitation of the current analysis was the retrospective nature of our study and a small sample size. Although >600 patients underwent TEER at our center during the study's period, only 134 had comprehensive assessment of their LVEF intraoperatively before and after TEER, which may introduce a selection bias. Hence, these results should be considered hypothesis generating and need to be validated in further studies. During TEER procedures, the results of preprocedural and postprocedural measuring of LVEF using transesophageal echocardiography may be influenced by general anesthesia and are not routinely conducted. Therefore, it is advisable to contemplate protocol modifications to ensure the collection of more comprehensive and accurate data. With additional data, further analyses can be conducted to investigate the mechanisms of subgroups such as functional mitral regurgitation and degenerative mitral regurgitation, as well as their impacts on prognosis.

## Sources of Funding

None.

## Disclosures

None.
